# Persistent Abdominal Pain as Rare Complication of Duodenal Perforation From an Inferior Vena Cava Filter

**DOI:** 10.7759/cureus.13168

**Published:** 2021-02-06

**Authors:** Walid Khan, Wei Zhang, Virginia Clark

**Affiliations:** 1 Department of Internal Medicine, University of Florida, Gainesville, USA; 2 Department of Gastroenterology and Hepatology, University of Florida, Gainesville, USA

**Keywords:** ivc filter, duodenum, perforation, chronic abdominal pain

## Abstract

Deep vein thrombosis (DVT) continues to be a significant source of morbidity for surgical patients. Inferior vena cava (IVC) filter placement is indicated for DVT in patients who have contraindications to anticoagulation or anticoagulation failure. Over the last decade, there is an exponential increase in IVC filter placement with increased complications reported. These include IVC penetration, IVC occlusion, insertion complication and filter migration. We report a rare case of symptomatic duodenal perforation by an IVC filter migration. This case illustrates that even though IVC migration and perforation is a rare complication, it should be recognized as a potential cause for gastrointestinal (GI) symptoms in these patients.

## Introduction

Inferior vena cava (IVC) filters came to be widely used in the 1970s for the prevention of pulmonary emboli. The only accepted indication for placement has been patients with venous thromboembolism (VTE) who have contraindications to anticoagulation, failure of anticoagulation, or complications to the anticoagulation they were on previously. However, with increased ease and the relative safety of the procedure, more filters are being used for patients that are considered high risk for thromboembolism especially after trauma [1]. From 1998 to 2005, it was seen that the rate of placement for prophylactic measures increased by 157% [2]. Despite it being such a common procedure, studies have shown that IVC filters do not have a statistically significant effect in reducing pulmonary embolism rates [3]. However, the filters have significant risks with studies show that patients had a higher incidence rate of deep vein thrombosis (DVT) after receiving an IVC filter since the underlying predisposition to thrombosis does not lessen with the placement of a filter [4]. One of the rare, later complications of IVC filter placement is migration into surrounding structures including the duodenum which can present with abdominal pain, gastrointestinal (GI) bleeding, or other nonspecific GI symptoms [5]. We present a patient with recurrent abdominal pain following IVC migration into the duodenum.

The case was previously presented at ACG Annual Meeting 2019 in San Antonio, Texas

## Case presentation

**Figure 1:**
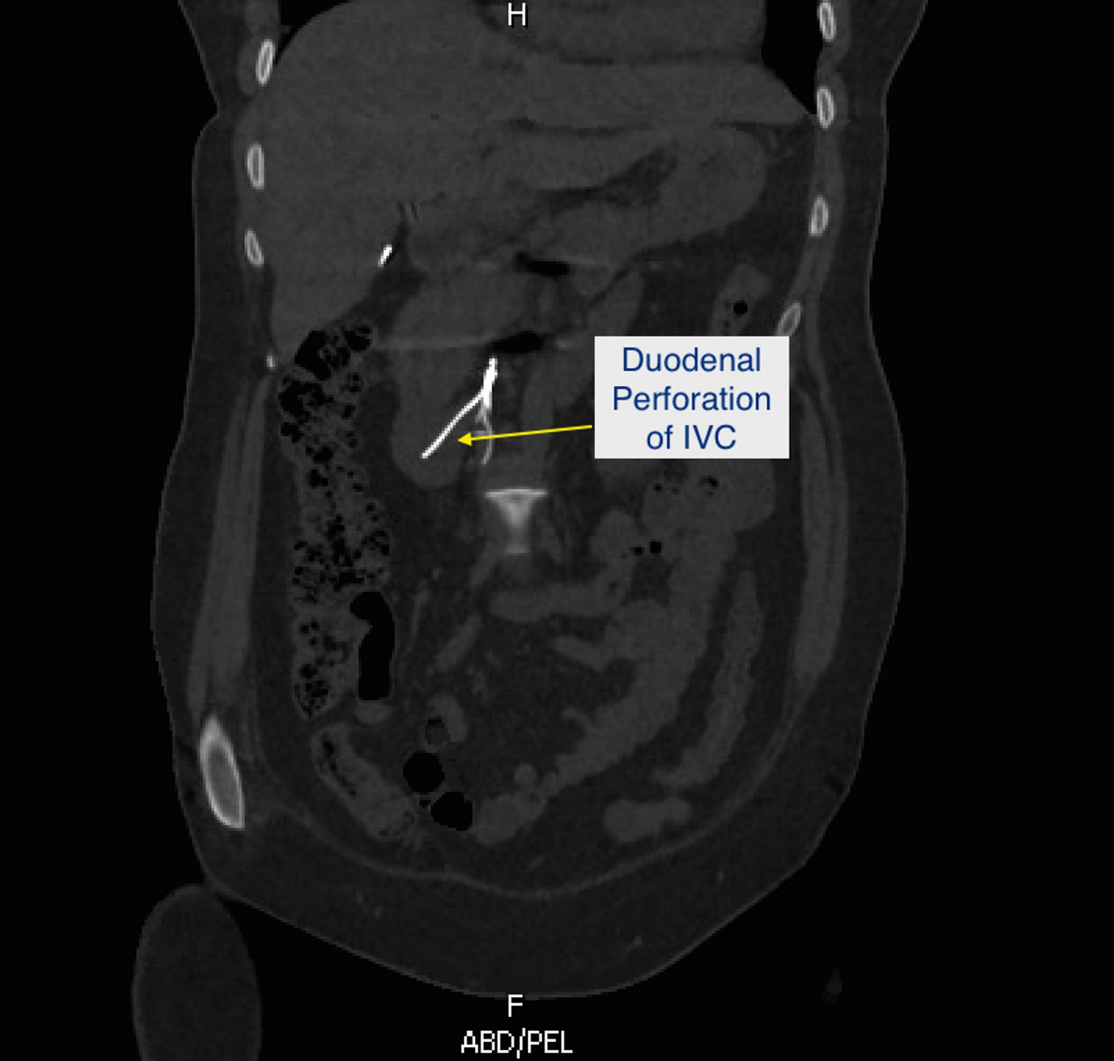
CT scan showing IVC filter penetration into duodenum. CT: computed tomography; IVC: inferior vena cava.

**Figure 2:**
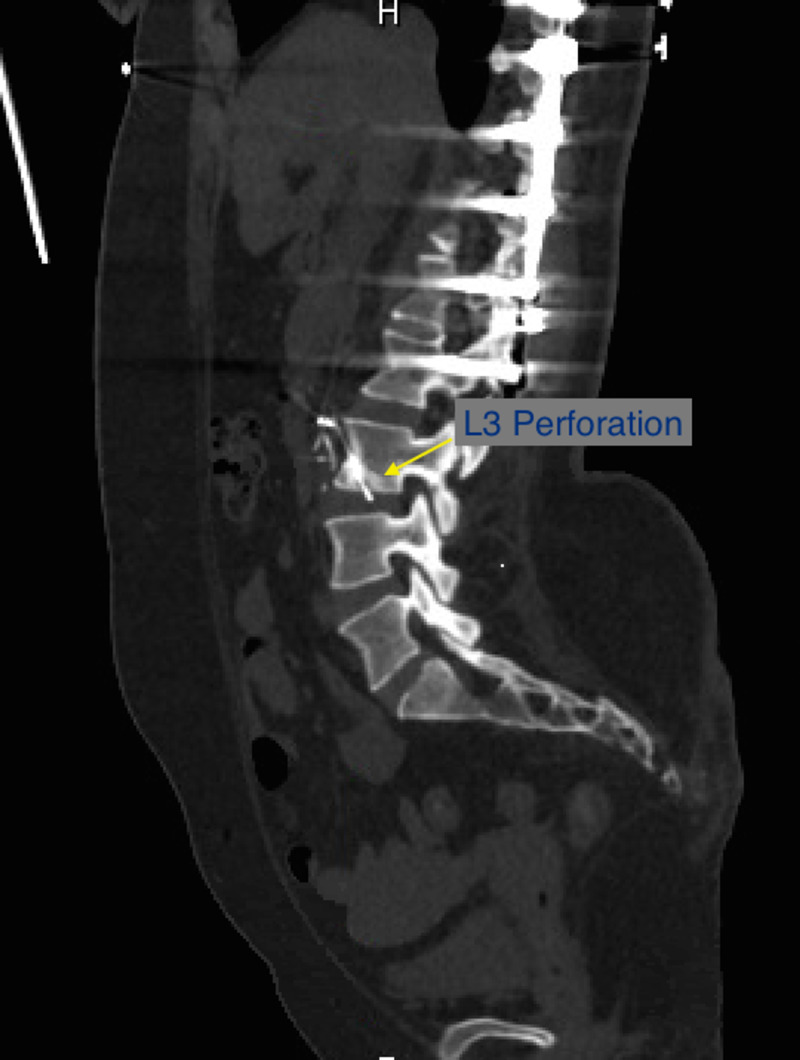
IVC filter penetrating into L3 vertebra. IVC: inferior vena cava.

A 33-year-old woman with a history of T11 paraplegia secondary to a motor vehicle collision, recurrent lower extremity thrombi on warfarin, and placement of a Cook Celect IVC filter in 2008 by vascular surgery as a prophylactic measure following neurosurgical intervention after MVC presented to the emergency department for the 10th time in a one-year period for acute worsening of chronic epigastric pain. She described the pain as an intermittent sharp pain that was more frequent at nighttime while lying down with no provoking or alleviating factors. Review of systems was negative outside the abdominal pain with patient denying any diarrhea, hematochezia, hematemesis, nausea, vomiting. On examination, there was tenderness to palpation in the epigastric and right upper quadrant of her abdomen with mild guarding with no distension or rigidity noted. Labs including complete blood count and basic metabolic panel were unremarkable. Computed tomography (CT) scan showed a longstanding IVC occlusion with multiple collaterals and displaced IVC filter into duodenum and into the L3 vertebral body (Figures 1, 2).

 

**Figure 3:**
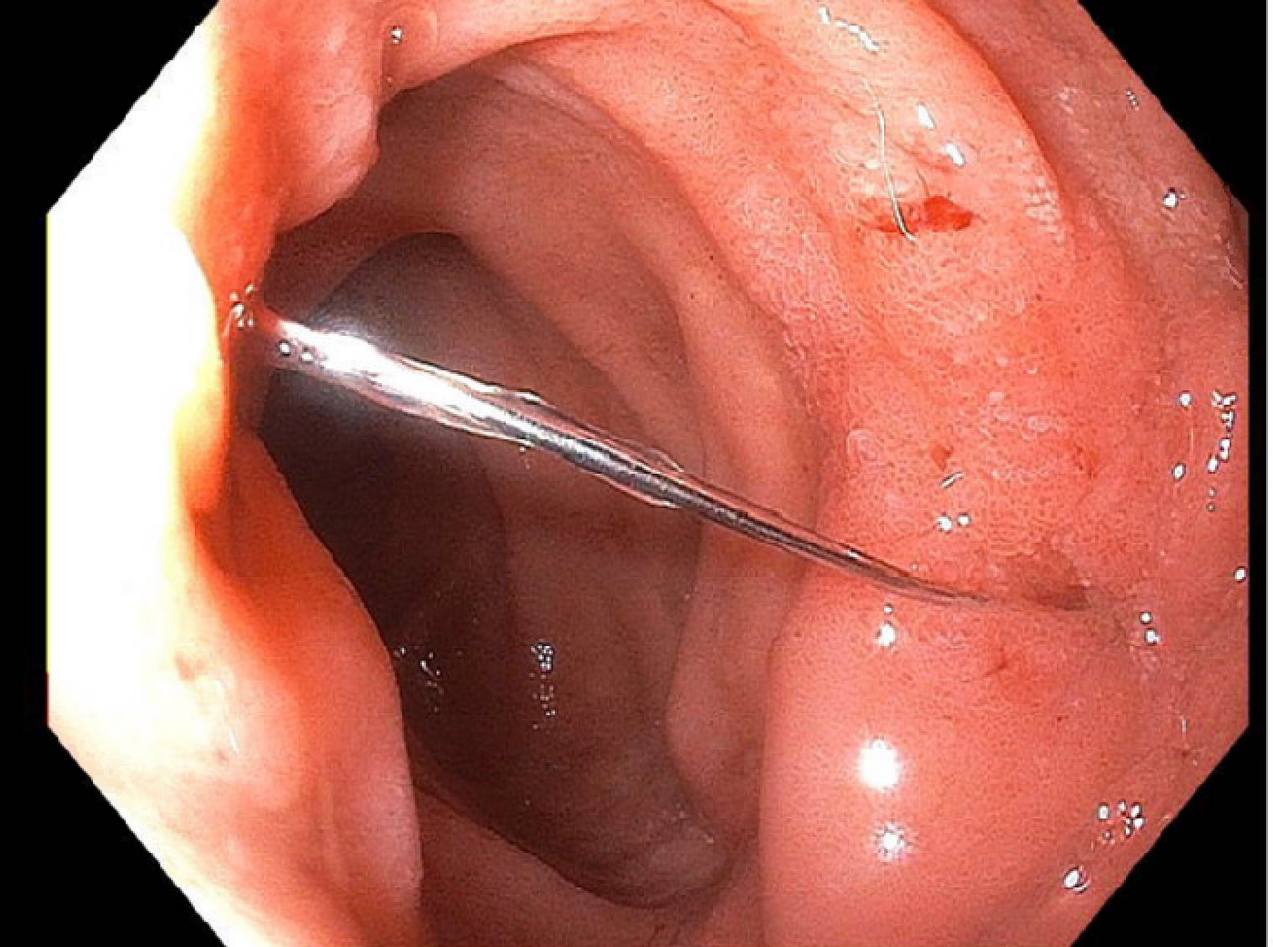
IVC strut penetrating duodenum. IVC: inferior vena cava.

An esophagogastroduodenoscopy (EGD) was done showing prong migration to the second portion of the duodenum (Figure 3). This was distal to the ampulla and penetrating the duodenal wall with the tip embedding the opposite wall. There was no ulceration or bleeding at the site of penetration. Rat tooth forceps were used to displace the tip that was embedded in the opposite wall

Due to multiple workups otherwise being negative for her abdominal pain with EGD and imaging findings consistent with duodenal perforation, vascular surgery was consulted and an elective surgery with open IVC filter extraction with duodenal and caval repair was scheduled.

## Discussion

IVC filters were first approved in the 1970s by the Food and Drug Administration (FDA) to decrease the risk of pulmonary emboli in patients where anticoagulation was not able to be used due to either contraindications or ineffectiveness. With off-label indications for placement such as prophylaxis in trauma patients and increased ease of placement, the frequency has increased. With this, an increased prevalence of complications has also been noted [1]. This increased observed prevalence of complications could partly be due to the ease of obtaining CT scans or EGD in present times allowing easier diagnosis. These complications also increase in frequency with the time that the filters have been in place so it is likely that these filters were not in place long enough for these complications to be noted [6]. IVC filters are meant to be a temporary measure with the FDA recommending that they be removed within 25-54 days. Due to a variety of factors, retrieval is often not even attempted with one large study estimating the overall retrieval rate to be around 34% despite a success rate of 80%-90% [7]. Much of this is due to loss of follow-up, but additional risk factors were mostly related to life expectancy or the root cause such as DVT, bleeding, or PE being unresolved [8]. One effective method of ensuring removal has been increasing physician education and patient programs ensuring regular follow-up; with these methods, 75% of temporary filters are removed [9].

Potential complications of IVC filter placement include DVTs, IVC thrombosis, IVC migration, perforation of either arterial or venous vessels, or perforation of surrounding structures [10]. Perforation has been reported to include structures such as the duodenum, aorta, portal veins, diaphragm, large intestine, and retroperitoneum. A systematic review by Jia et al. showed 9002 patients with IVC filters in cited studies with 322 cases of organ penetration with the duodenum being the most commonly involved organ (123 cases). Only 8% of the total 322 cases were noted to be symptomatic [11]. Because symptomatic duodenal perforation is a rare complication, there are no guidelines regarding diagnostic or treatment strategies. However, all current case reports on the topic of duodenal perforation have shown that the filters were placed more than 10 months before the onset of symptoms [6]. This further emphasizes the importance of a system to ensure timely removal of IVC filters such as education for physicians or automatically scheduled follow-up. Another factor that appears to play a role in IVC filter complications is the geometry and brand of the filter placed. Our patient received a Celect filter which has a conical shape that is more commonly associated with a high rate of penetration versus other types of IVC filters [11-13]. However certain brands of conical filters seem to have a much lower rate of penetration associated with their use when compared with the Celect filter [14]. Standardized studies need to be done comparing different shapes and brands to further analyze the role of these factors. The Celect filter is a temporary filter and this just further stresses the importance of staying vigilant in regards to removing these temporary IVC filters when possible.

The two methods of removal in the setting of perforation are open and endovascular repair. A systematic review article by Malgor and Labropulos showed in the setting of symptomatic duodenal perforation by IVC filters, 20/21 filters were removed by open surgery. 19/20 of these filters were placed between 7 and 180 months before patient presentation showing the prolonged time course between placement and symptoms secondary to perforation [6]. In the systematic review by Jia et al. describing all complications requiring removal, 4/83 IVC filters were removed using endovascular retrieval [11]. However, endovascular retrieval is increasingly being recognized as a safe option saving the patient from an invasive open repair in selective patients [15-17].

## Conclusions

This case report emphasizes the importance of keeping IVC filter migration and penetration on the differential for a myriad of GI symptoms. Our patient had her temporary Celect filter in place for 11 years despite long term rivaroxaban therapy and no further evidence of DVT. Filter removal could have been considered to avoid further complications. With increasing evidence showing that abdominal pain and GI bleeding can be secondary to duodenal penetration of an IVC filter, clinicians should have a high suspicion for this complication which can be confirmed by endoscopy. Based on the success of physician education and programs for patient follow-up for removal, similar ideas should be instituted across systems where there is low IVC filter retrieval to help prevent complications such as migration which is most likely to occur with the passage of time.
